# Recent Developments and Evolving Therapeutic Strategies in *KMT2A* ‐Rearranged Acute Leukemia

**DOI:** 10.1002/cam4.70326

**Published:** 2024-10-21

**Authors:** Lei Yin, Lin Wan, Youjian Zhang, Shenghao Hua, Xuejun Shao

**Affiliations:** ^1^ Department of Clinical Laboratory Children's Hospital of Soochow University Suzhou China; ^2^ Department of Pediatrics The First Affiliated Hospital of Shandong First Medical University & Shandong Provincial Qianfoshan Hospital Jinan China

**Keywords:** *KMT2A*‐rearranged acute leukemia, lymphoblastic leukemia, myeloid leukemia, therapy

## Abstract

**Background:**

Rearrangements of the histone‐lysine‐N‐methyltransferase *(KMT2A)*, previously referred to as mixed‐lineage leukemia *(MLL),* are among the most common chromosomal abnormalities in patients with acute myeloid leukemia (AML) and acute lymphoblastic leukemia (ALL), involving numerous different fusion partners. *KMT2A*‐rearranged (*KMT2A*‐r) leukemia is characterized by a rapid onset, aggressive progression, and significantly worse prognosis compared to non‐*KMT2A*‐r leukemias. Even with contemporary chemotherapeutic treatments and hematopoietic stem cell transplantations (HSCT), patients with *KMT2A*‐r leukemia typically experience poor outcomes and limited responses to these therapies.

**Objectives:**

This review aims to consolidate recent studies on the general gene characteristics and associated mechanisms of *KMT2A‐*r acute leukemia, as well as the cytogenetics, immunophenotype, clinical presentation, and risk stratification of both *KMT2A‐*r‐AML and *KMT2A*‐r‐ALL. Particularly, the treatment targets in *KMT2A*‐r acute leukemia are examined.

**Methods:**

A comprehensive review was carried out by systematically synthesizing existing literature on PubMed, using the combination of the keywords ‘*KMT2A*‐rearranged acute leukemia’, ‘lymphoblastic leukemia’, ‘myeloid leukemia’, and ‘therapy’. The available studies were screened for selection based on quality and relevance.

**Conclusions:**

Studies indicate that *KMT2A* rearrangements are present in over 70% of infant leukemia cases, approximately 10% of adult AML cases, and numerous instances of secondary acute leukemias, making it a disease of critical concern to clinicians and researchers alike. The future of *KMT2A*‐r acute leukemia research is characterized by an expanding knowledge of the disease's biology, with an emphasis on personalized therapies, immunotherapies, genomic advancements, and innovative therapeutic combinations. The overarching aim is to enhance patient outcomes, lessen the disease burden, and elevate the quality of life for those affected. Ongoing research and clinical trials in this area continue to offer promising opportunities for refining treatment strategies and improving patient prognosis.

## 1 Introduction

Histone‐lysine‐N‐methyltransferase‐rearranged (*KMT2A*‐r) acute leukemia, commonly known as mixed lineage leukemia (*MLL*), is a distinct subtype of acute leukemia marked by genetic abnormalities in the *KMT2A* gene (also known as *ALL‐1, HRX*, or *HTRX*). In patients, both adult and pediatric, this disease has been linked to a poor prognosis [1]. Several studies have explored key aspects of *KMT2A*‐r acute leukemia, including its genetic basis, clinical implications, and treatment options [2, 3, 4, 5]. A specific genetic rearrangement occurs in *KMT2A*‐r acute leukemia, most commonly represented by the translocation t(4;11)(q21;q23) [1, 6]. This genetic alteration fuses the *KMT2A* gene with another gene, often the *AF4* gene, creating a fusion protein that disrupts the normal function of *KMT2A*. This disruption leads to uncontrolled cell growth and the development of leukemia [7, 8]. Regarding the clinical implications of this condition, *KMT2A*‐r acute leukemia is linked to negative clinical outcomes. Individuals with this subtype often have a more aggressive disease course and a greater chance of relapse as compared to other kinds of leukemia [1, 9]. It is more prevalent in infants and very young children, accounting for a significant portion of infant leukemia cases. This genetic abnormality is less common in adults but still challenging to treat [10].

*KMT2A*‐r acute leukemia typically involves genetic testing to identify the specific *KMT2A* rearrangement. Different molecular techniques can be used to achieve this, such as fluorescence in situ hybridization (FISH) and polymerase chain reaction (PCR) [6]. Detecting this genetic alteration is crucial for both confirming the diagnosis and guiding treatment decisions. Managing *KMT2A*‐r acute leukemia is complex and often requires intensive therapy. Standard treatment approaches may include chemotherapy, targeted therapies, supportive care, and transplanting stem cells. Researchers have recently investigated novel therapies, including epigenetic and immunotherapy approaches, to provide better outcomes for people suffering from this high‐risk subtype [11]. The prognosis for *KMT2A*‐r acute leukemia remains challenging due to its aggressive nature. However, advancements in treatment and ongoing research are gradually improving survival rates and long‐term outcomes. Early detection and appropriate therapeutic interventions are key factors in increasing the chances of remission and long‐term survival for patients with *KMT2A*‐r leukemia.

Considering the fact that *KMT2A*‐r leukemia is a complex disease with evolving research, this review aims to synthesize and update our knowledge in this field, providing a comprehensive overview of the latest findings and understanding of the condition. We will explore the general characteristics, clinical presentations, and treatment options of *KMT2A*‐r leukemia. This will contribute to understanding clinical implications, including diagnosis, prognosis, and treatment strategies, which is essential for healthcare professionals and researchers.

## 2 General Gene Characteristics and Associated Mechanisms

For proper hematopoiesis to occur, the *KMT2A* gene is essential. It regulates gene expression by modifying chromatin structure. In *KMT2A*‐r acute leukemia, chromosomal rearrangements disrupt the *KMT2A* gene, leading to fusion with partner genes, including *AF9, ENL, AF4*, *AFF6, AF10, ELL*, and *KMT2A‐PTDS*, which together are responsible for most *KMT2A* fusions [12, 13]. In effect, the fusion of 11q23 with greater than 100 distinct partner genes occurs in *KMT2A*‐r leukemias, resulting in various KMT2A fusion proteins. These highly complex fusion events can have different functional consequences [11, 14]. The partner genes that fuse with the *KMT2A* gene vary in different cases of leukemia, and the specific fusion partner can impact the disease's characteristics and prognosis. Understanding these partner genes is crucial for studying the pathogenesis of *KMT2A*‐r leukemias and developing targeted therapies.

The characteristic changes in the *KMT2A* gene are associated with several oncogenic mechanisms, including the generation of oncogenic fusion proteins, epigenetic dysregulation, *homeobox A* (*HOXA*) cluster gene activation, and aberrant self‐renewal, contributing to the poor prognosis and therapeutic challenges that characterize *KMT2A*‐r acute leukemia. Combining *KMT2A* with partner genes creates oncogenic fusion proteins that have a disrupted KMT2A domain, impairing its normal regulatory functions [15, 16]. Moreover, *KMT2A*‐r leukemia is linked to profound epigenetic dysregulation, such as histone acetylation, histone methylation, and DNA methylation [17]. The fusion proteins affect the modification of histones and chromatin structure, leading to abnormal gene expression patterns [14]. This deregulation contributes to leukemia's development and provides targets for alternative strategies for leukemia treatment [17, 18].

**FIGURE 1:**
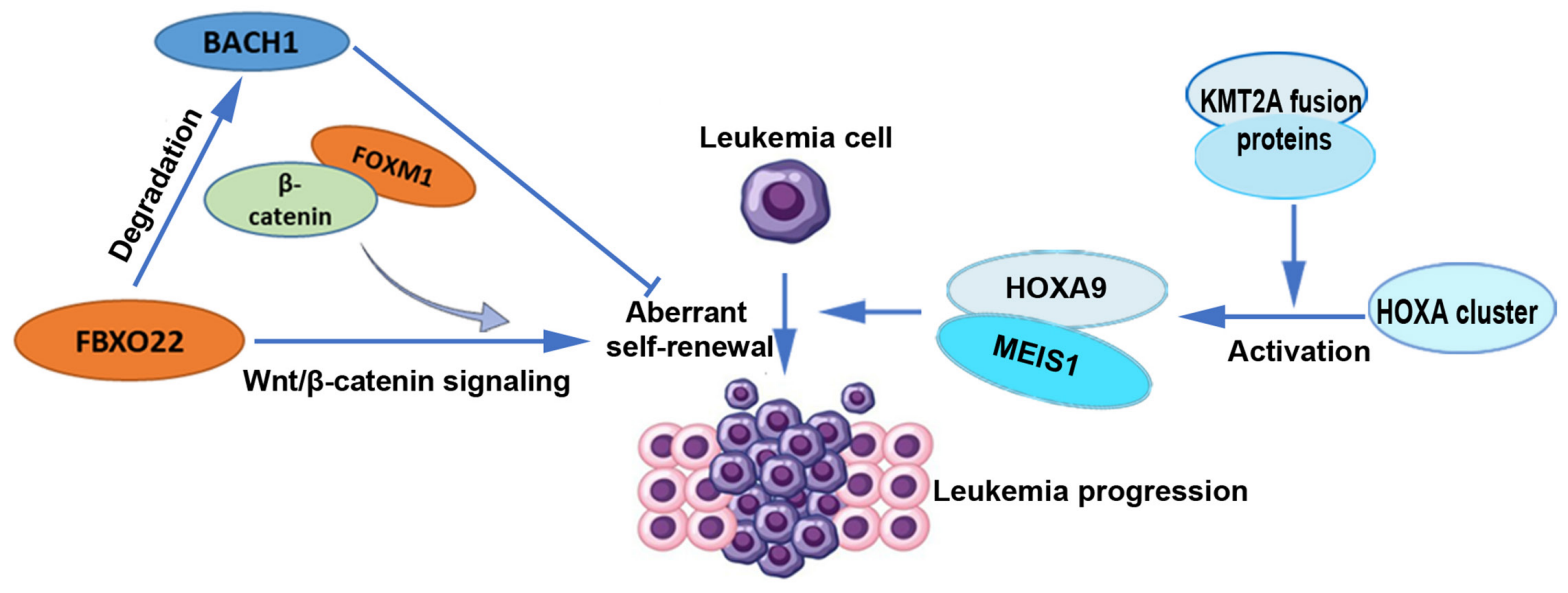
HOXA9/MEIS1 and FBXO22 promotion of *KMT2A*‐r acute leukemia. One of the main ways that *KMT2A*‐r acute leukemia works is by activating *HOXA* cluster genes, especially *HOXA9* and *MEIS1*. The fusion proteins that are created when the *KMT2A* gene breaks and fuses with another gene make these genes more active. *KMT2A*‐r acute leukemias often have the ability to renew themselves. FBXO22 protein helps *KMT2A*‐r acute leukemia cells to renew themselves by increasing the degradation of BACH1. Since an elevated expression of BACH1 inhibits the cancer process, low levels in cells are associated with self‐renewal and lack of cell differentiation. By binding to and stabilizing β‐catenin, FOXM1 aids in maintaining the activity of the Wnt/β‐catenin signaling pathway, which encourages leukemia stem cells to self‐renew.

One of the key mechanisms of *KMT2A*‐r acute leukemia involves the activation of *HOXA* cluster genes, particularly *HOXA9* and *MEIS1* [19] (Figure 1). The fusion proteins can increase these genes' expression, promoting leukemogenesis. Therefore, in *KMT2A*‐r acute leukemia, downregulation of the *HOXA* and *MEIS1* genes may decrease proliferation and hinder engraftment [20]. Since *KMT2A*‐r acute myeloid leukemia (*KMT2A*‐r‐AML) is characterized by the overexpression of *HOXA/MEIS1/PBX3* homeobox genes, tackling the interactions of MEIS1/PBX3 may offer a promising therapeutic approach for treating these AML subtypes [21]. Another key mechanism is attributed to *KMT2A*‐r leukemias often exhibiting enhanced self‐renewal capacity, which allows leukemic cells to proliferate and evade normal regulatory mechanisms. For example, F‐box protein 22 (FBXO22) promotes *KMT2A*‐r‐AML aberrant self‐renewal by enhancing the degradation of BACH1 (BTB domain and CNC homolog 1) [22]. Moreover, it has been demonstrated that *FOXM1* (Forkhead Box M1), a known transcription factor that stimulates cell proliferation in a range of cancer cells, is necessary for the quiescence, self‐renewal, and survival of leukemia stem cells (LSCs) transformed by MLL‐AF9 in vivo. Mechanistically, elevated FOXM1 expression stimulates the Wnt/β‐catenin signaling pathways by attaching itself to the β‐catenin protein and preventing its breakdown., thereby preserving LSC quiescence and promoting LSC self‐renewal in *KMT2A*‐r‐AML [23] (Figure 1). Studies such as these demonstrate how important these mechanisms are to *KMT2A*‐r‐AML and present potential therapeutic targets for exploration.

## 3 *KMT2A* ‐Rearranged Acute Lymphoblastic Leukemia (KMT2A‐r‐ALL)

Genetic rearrangements of *KMT2A* with partner genes, producing KMT2A fusion proteins and driving *KMT2A*‐r acute lymphoblastic leukemia (*KMT2A*‐r‐ALL) [6]. More than 100 partner genes have been identified, with the most prevalent ones being *AFF1* (formerly *AF4*; ∼56.5%), *MLLT1* (previously *ENL*, ∼18.5%), and *MLLT3* (previously *AF9*; ∼11.8%) [6]. *KMT2A*‐r‐ALL is prevalent in newborns under 1 year old. A rare yet extremely aggressive form of juvenile leukemia known as *KMT2A*‐r infant ALL (*KMT2A*‐r iALL) is known for its poor prognosis and challenging treatment [24]. It is associated with unfavorable clinical outcomes. The genetic landscape of *KMT2A*‐r‐ALL is complex, with distinct somatic mutations and genetic alterations that contribute to the disease's aggressiveness [6, 11].

### 3.1 Cytogenetics and Immunophenotype

**FIGURE 2:**
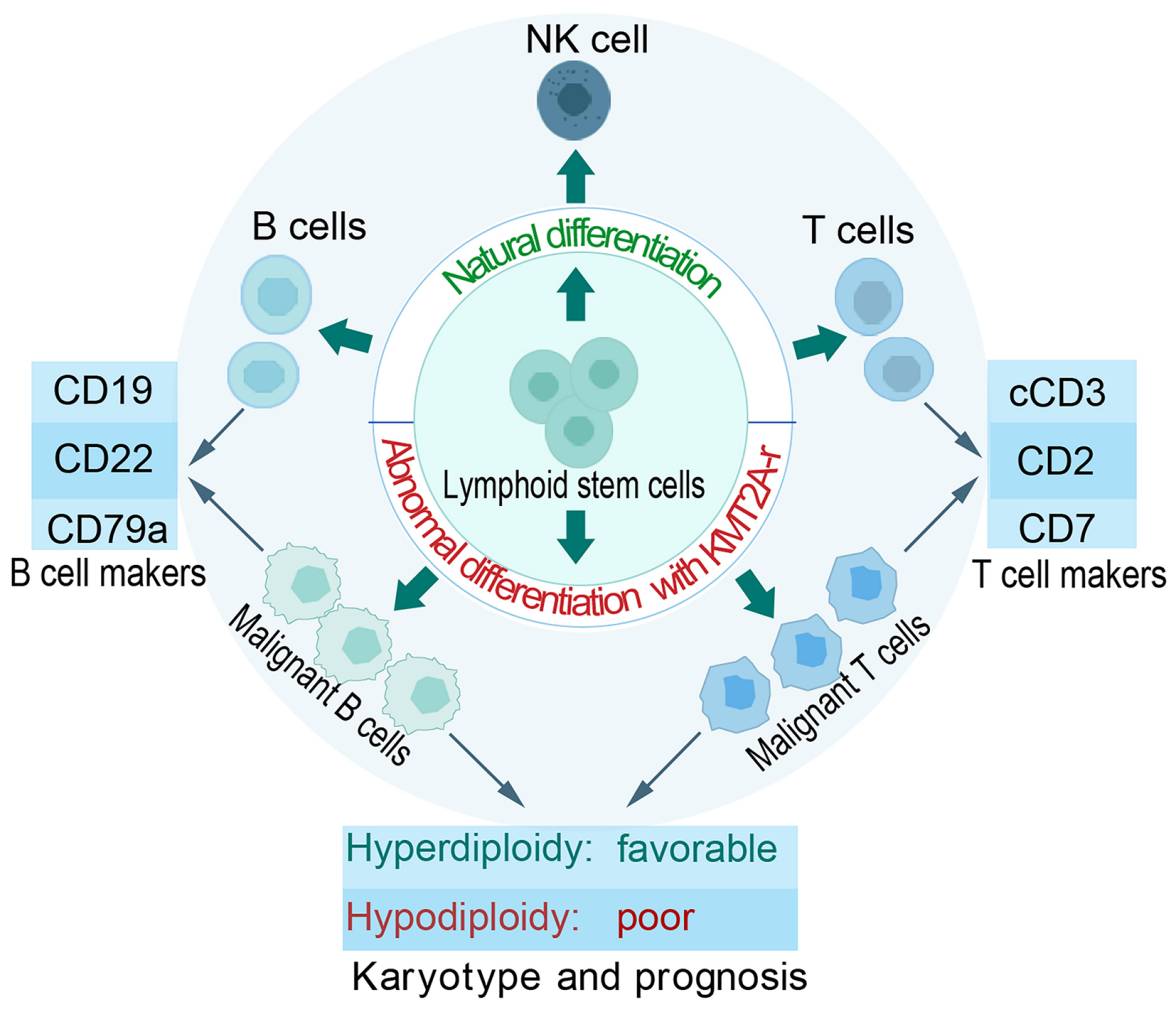
Cytogenetics and immunophenotype of *KMT2A*‐r‐ALL. *KMT2A*‐r‐ALL mainly affects lymphocytes, specifically B cells and T cells. The cancer cells grow uncontrollably and disrupt the production of normal lymphocytes. This leads to a buildup of immature lymphocytes. In *KMT2A*‐r‐ALL, the karyotype often reveals either hyperdiploidy or hypodiploidy. A favorable prognosis is typically linked to hyperdiploidy with more than 50 chromosomes, while a poor prognosis is connected to hypodiploidy with ≤ 43 chromosomes. The leukemic cells express markers associated with lymphoid lineage differentiation, such as CD19, CD79a, CD22 for B‐ALL, and CD7, CD2, and cCD3 for T‐ALL.

*KMT2A*‐r‐ALL, as a subtype of acute lymphoblastic leukemia, predominantly affects lymphoid cells. These cells, including B‐cells, NK cells, and T‐cells, become malignant and disrupt the production of normal lymphocytes, resulting in an increased production of immature lymphoid cells [9]. In *KMT2A*‐r‐ALL, the karyotype often reveals either hyperdiploidy or hypodiploidy (Figure 2). These cytogenetic abnormalities are associated with the disease and can influence the prognosis [25]. *KMT2A*‐r‐ALL typically exhibits a lymphoid immunophenotype. The leukemic cells express markers associated with lymphoid lineage differentiation, such as CD19, CD79a, and CD22 for B‐ALL, and CD7, CD2, and cytoplasm CD3 (cCD3) for T‐ALL (Figure 2). These markers are consistent with the B‐cell or T‐cell origin of the leukemia [26]. Unlike typical precursor B‐ or T‐cell ALL, *KMT2A*‐r‐ALL often lacks expression of terminal deoxynucleotidyl transferase (TdT), a marker typically present in lymphoblasts. This loss of TdT expression can help differentiate *KMT2A*‐r‐ALL from other subtypes of ALL [6, 26]. It is essential to note that the specific partner gene involved in the KMT2A rearrangement can influence the immunophenotype and clinical features of *KMT2A*‐r‐ALL. Therefore, a comprehensive evaluation of both cytogenetics and immunophenotype is crucial for diagnosis and risk stratification.

### 3.2 Risk Stratification and Clinical Presentation

*KMT2A*‐r‐ALL is also aggressive and may occur more commonly in children. Patients with *KMT2A*‐r‐ALL may present with symptoms related to the overproduction of lymphoid cells, including enlarged lymph nodes and spleen, and a higher chance of central nervous system involvement [9]. This necessitates careful monitoring and prophylactic treatment to prevent or manage CNS disease. In addition, patients often present with rapidly progressing symptoms, including fatigue, fever, bone pain, and bleeding tendencies. Some cases of *KMT2A*‐r‐ALL may exhibit features of mixed phenotype acute leukemia (MPAL), defined by the simultaneous expression of myeloid and lymphoid markers. This hybrid immunophenotype can make diagnosis and treatment challenging [1, 26].

Although *KMT2A*‐r‐ALL is known for having a worse prognosis than non‐*KMT2A*‐r leukemias, there is significant heterogeneity in outcomes. Several factors influence risk stratification, including age, specific MLL fusion partner, response to treatment, and CNS disease. It is reported that younger patients, particularly babies suffering from *KMT2A*‐r‐ALL, frequently have a worse prognosis as compared to adults and older kids. Moreover, the partner gene involved in the *KMT2A* rearrangement can influence prognosis. Some fusion partners may be associated with a more favorable outcome than others [26, 27]. The response to initial treatment is a crucial factor in risk stratification. Patients with a slow or poor response may have a greater chance of relapse. As was previously indicated, the existence of CNS involvement upon diagnosis is a risky factor, and patients with this feature may require more intensive therapy [28]. Ongoing research is exploring targeted therapies and novel treatment approaches for *KMT2A*‐r‐ALL, intending to improve outcomes and reduce relapse rates.

## 4 *KMT2A* ‐Rearranged Acute Myeloid Leukemia ( *KMT2A* ‐r‐AML)

**FIGURE 3:**
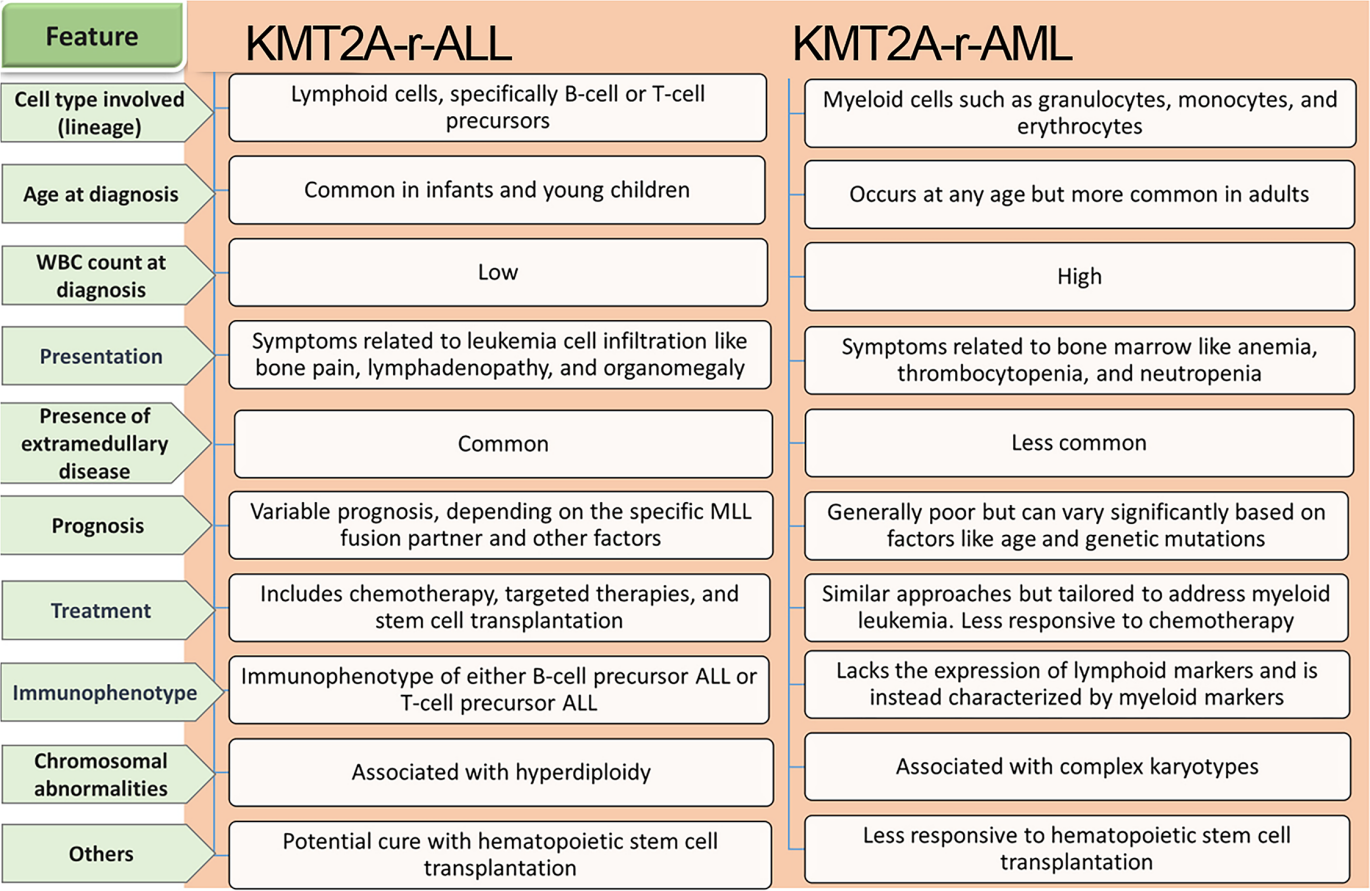
Key distinctions between *KMT2A*‐r‐AML and *KMT2A*‐r‐ALL. The differences between these two distinct *KMT2A*‐r acute leukemias can be assessed based on elements such as lineage, common age range at diagnosis, and the number of white blood cells (WBC) at diagnosis and presentation. Other factors include prognosis, treatment, immunophenotype, and chromosomal abnormalities.

*KMT2A*‐r‐ALL and *KMT2A*‐r‐AML are two distinct subtypes of leukemia, each distinguished by *KMT2A* rearrangements on a genomic level. Like *KMT2A*‐r‐ALL, *KMT2A*‐r‐AML is determined by chromosomal translocations involving the *KMT2A* gene, found on chromosome 11q23. Moreover, Both *KMT2A*‐r‐AML and *KMT2A*‐r‐ALL are associated with poor prognoses, but the specific prognostic factors and outcomes can vary depending on variables such as genetic subtypes, age, and how they react to treatment. Despite the challenges, treatment strategies and advances continue to improve some patients' survival rates [26]. Key differences exist in their cell lineage, clinical characteristics, treatment strategies, and prognostic implications. *KMT2A*‐r‐AML specifically manifests as an acute myeloid leukemia subtype, often exhibits a complex genetic landscape, and the specific *KMT2A* fusion partner genes can vary, contributing to the heterogeneity of the disease [26]. Figure 3 presents the key distinctions between *KMT2A*‐r‐ALL and *KMT2A*‐r‐AML [29, 30, 31].

### 4.1 Cytogenetics and Immunophenotype

*KMT2A*‐r‐AML is a subtype of acute myeloid leukemia that primarily affects cells of the myeloid lineage, which causes the bone marrow to produce an excessive amount of immature myeloid cells [11]. *KMT2A*‐r‐AML often presents with complex karyotypes, indicating multiple chromosomal abnormalities. These complex karyotypes are a hallmark of *KMT2A*‐r‐AML and contribute to the disease's aggressiveness [11, 32]. In addition to *KMT2A* rearrangements, *KMT2A*‐r‐AML may carry cooperating genetic alterations in genes such as *FLT3, NPM1*, and *IDH1/2*, further contributing to the disease's pathogenesis and clinical behavior [33, 34]. The immunophenotype of *KMT2A*‐r‐AML typically shows the manifestations of myeloid markers such as CD33, CD13, and MPO (myeloperoxidase) [35]. The expression of these markers is consistent with the myeloid lineage differentiation characteristic of AML. *KMT2A*‐r‐AML cells may also exhibit hematopoietic stem and progenitor cell markers, such as CD34 and CD117 (c‐kit) [35]. The presence of these markers suggests that the leukemic cells originate from an immature hematopoietic precursor population. These immunophenotypic and cytogenetic features play crucial roles in diagnosing and classifying AML and can guide treatment decisions [33].

### 4.2 Clinical Presentation and Risk Stratification

*KMT2A*‐r‐AML is characterized by an abrupt onset, rapid advancement, and an extremely bad prognosis in contrast to non‐*KMT2A*‐rearranged cases. The swift multiplication of myeloid cells in AML can lead to symptoms such as tiredness, anemia, and an increased risk of infections [1, 36]. Other clinical symptoms include breathing difficulties, bleeding and bruising easily, fever, bone pain, bone marrow failure, headaches, night sweats, and weight loss. The prognosis of *KMT2A*‐r‐AML is dependent on variables like the patient's age, specific *KMT2A* fusion partner, other chromosomal abnormalities, and overall health [31]. The European LeukemiaNet (ELN) risk stratification system classifies *KMT2A*‐r‐AML into three risk categories: favorable, intermediate, and poor. In the favorable risk group, there is the translocation of t(9;11)(p22;q23) in patients and no other chromosomal abnormalities. These patients are typically young and have a decreased number of white blood cells at diagnosis. Patients with other *KMT2A* rearrangements or chromosomal abnormalities, in addition to *KMT2A* rearrangements, have an intermediate prognosis. These patients are typically older and have a higher white blood cell count at diagnosis [29]. *KMT2A‐AFF1* (*MLL‐AF4*) gene fusion presents symptoms such as hepatosplenomegaly, coagulopathy, and severe organ infiltration. Affected individuals who do not experience remission with standard chemotherapy are considered high risk and may require more aggressive treatment approaches, such as the transplantation of hematopoietic stem cells. These individuals may be candidates for stem cell transplantation, innovative therapies, or experimental treatments.

## 5 Current and Exploratory Therapeutic Targets

*KMT2A*‐r acute leukemia therapeutic targets have been the subject of intensive research due to the aggressive nature of these leukemias. These therapies can be grouped based on the key component of the *KMT2A*‐r acute leukemia microenvironment being targeted.

### 5.1 Targeting KMT2A Fusion Proteins

Central to *KMT2A*‐r leukemia are KMT2A fusion proteins, which are the product of chromosomal translocations. Research has focused on targeting these fusion proteins directly. Small molecule inhibitors and monoclonal antibodies are being explored to disrupt the function of KMT2A fusion proteins. For example, studies have explored the target of Menin‐KMT2A [2, 32] and KMT2A‐AFF1 (MLL‐AF4) [13, 37] interactions. The Menin protein is crucial for KMT2A to regulate the expression of pertinent target genes, as well as facilitate the high‐affinity interactions occurring between the amino termini of KMT2A proteins. Menin proteins are also required to facilitate KMT2A fusion protein oncogenic transformation [38]. Thus, for *KMT2A*‐r leukemias, one possible treatment approach is to hinder the Menin‐KMT2A interaction. According to recent literature, the FDA has designated Menin‐KMT2A interaction inhibitors like SNDX‐5613 and KO‐539 as orphan drugs for the treatment of refractory/relapsed leukemia and AML, respectively [32, 39]. Relapse is associated with a lineage flip from ALL to AML, which can cause chemotherapy and immunotherapy resistance, leading to poor clinical outcomes [7]. According to a study, *MLL‐AF4* cells exhibit increased expression of immunoproteasomes unique to lymphoid tissues and are responsive to pharmacologically relevant doses of the immunoproteasome inhibitor ONX‐0914. In *MLL‐AF4* ALL, the inhibitors ONX‐0914 and LU‐102 exhibit synergistic cytotoxicity, and ONX‐0914 markedly delays the development of mice with orthotopic ALL xenograft tumors [40]. Inhibitors to other regular fusions of KMT2A (MLL), such as MLL‐ENL, MLL‐AF10, MLL‐AF4, and MLL‐AF9, as well as their mode of action, have been explored [41].

### 5.2 Epigenetic Regulators

KMT2A fusion proteins often influence epigenetic regulation, leading to aberrant gene expression. Therapies targeting epigenetic modifiers, including DNA methyltransferase inhibitors and histone deacetylase (HDAC) inhibitors, aim to correct the epigenetic dysregulation seen in *KMT2A*‐r leukemia. Vorinostat and panobinostat are HDAC inhibitors that affect histone acetylation, whereas 5‐azacitidine and decitabine are used to target DNA methylation [42]. In a recent study, the authors explored the impact of the potent HDAC inhibitor, I1, and its potential mechanism on cells of *KMT2A*‐r‐AML and *KMT2A*‐r‐ALL. They found that I1 provokes *KMT2A*‐r‐AML and *KMT2A*‐r‐ALL cells to differentiate via epigenetic modification as it significantly inhibited the proliferation and the colony‐forming ability of the cancer cells by promoting cell differentiation, combined with the blockage of the cell cycle at the G0/G1 phase [43]. This offers an epigenetic regulator, such as I1, as an effective epigenetic drug to prevent differentiation block in *KMT2A*‐r acute leukemia patients, and it would be promising for treating acute leukemias. Another study observed reduced Eμ‐Myc lymphoma proliferation and differentiation in acute promyelocytic leukemia using the HDAC3 inhibitor RGFP96 (APL). In both in vitro and in vivo settings, genetic co‐depletion of Hdac1 and Hdac2 was pro‐apoptotic in Eμ‐Myc lymphoma, and this behavior was mimicked by the HDAC1/2‐specific agent RGFP233 [44]. It has been demonstrated that other HDAC inhibitors, such as I3 [45] and I13 [46], are strong chromatin‐remodeling agents that can overcome the differentiation block in AML patients, including those with t(8; 21) translocation or *KMT2A* rearrangements. These inhibitors may be a potent and specific therapy option for AML. In addition to vorinostat and panobinostat, other HDAC inhibitors approved by the FDA for hematological malignancies are romidepsin and belinostat, and they inhibit class II and/or class I HDACs, including HDAC1, 2, 3, and 6 [44].

By integrating into DNA, hypomethylating substances like decitabine and azacitidine lessen DNA hypermethylation. In cases of acute lymphoblastic leukemia with *KMT2A* rearrangements, decitabine significantly delays leukemic proliferation in vivo xenograft models and inhibits proliferation in vitro by provoking the arrest of cell cycle arrest in the G0/G1 phase and apoptosis [47]. These studies provide evidence of the potential of these epigenetic regulators in *KMT2A*‐r acute leukemias and warrant more clinical evaluation.

### 5.3 Transcriptional Dependencies

Transcriptional dependencies that arise from KMT2A fusions provide therapeutic opportunities. Inhibitors of specific transcriptional co‐activators or elongation factors that interact with KMT2A fusion proteins can disrupt leukemic transcriptional programs. Therapeutic elements such as bromodomain‐containing protein 4 (BRD4), disruptor of telomeric silencing 1‐like (DOT1L), and SET inhibitors have gained much research interest and have advanced into clinical trials. For example, the DOT1L inhibitor EPZ‐5676 has shown moderate clinical activity in *KMT2A*‐r acute leukemia clinical trials [41, 48]. It is also reported that small‐molecule inhibition or genetic disruption of DOT1L and BRD4 shows significant synergistic activity against *KMT2A*‐r leukemia cell lines [49]. Moreover, when made to work together, SIRT1 activators and DOT1L inhibitors exhibit increased antiproliferative action against *KMT2A*‐r leukemia cells [50]. In other studies, super elongation complex (SEC) inhibitors such as THZ1 and THZ531 serve as small molecules that target SEC, disrupting transcription elongation [51, 52]. Through apoptosis induction and cell proliferation inhibition, THZ1 exhibits anti‐tumor actions in a variety of cancers. In a study, AML cell viability was found to be decreased by THZ1, which also activated G0/G1 cell cycle arrest, induced apoptosis in a time‐ and dose‐dependent manner, downregulated the phosphorylation of CDK1 and CDK2, and decreased RNA Pol II phosphorylation at several serine sites [53]. The activation of CDKs, or cyclin‐dependent kinases, is involved in the unrestrained growth of tumor cells. Thus, CDK inhibitors such as CYC065 and THZ1 disrupt leukemic transcriptional programs by reducing CDKs, leading to suppressed invasion, induced viability loss due to the arrest of the G2/M cell cycle, decreased proliferation, and promoted apoptosis [54]. Together, these findings highlight the potential of these therapeutic agents to suppress tumor growth and promote the death of cells, warranting further clinical investigation.

### 5.4 FLT3 and Other Signaling Pathways

Aberrant activation of FLT3 and other signaling pathways is common in *KMT2A*‐r‐AML. Small molecule inhibitors targeting FLT3 and other kinases like JAK2 (Janus‐kinase 2) and PI3K (phosphoinositid‐3‐kinasen) have shown promise in preclinical studies. FLT3 inhibitors such as midostaurin and gilteritinib [55] target *FLT3* mutations seen in AML, while JAK2 inhibitors such as ruxolitinib [56] inhibit JAK2, which may be activated in *KMT2A*‐r leukemia. Moreover, PI3K inhibitors such as idelalisib and copanlisib have been documented [57]. The combined use of selective small‐molecule kinase inhibitors of FLT3 phosphorylation and Menin‐KMT2A inhibition results in a marked downregulation of phosphorylated FLT3 and downstream transcriptional genes of FLT3 signaling. Synergistic suppression of proliferation was triggered by this particular drug combination and increased apoptosis in *KMT2A*‐r leukemias with an FLT3 mutation as opposed to single‐drug therapy. It also reduced leukemia burden and prolonged survival in *KMT2A*‐r FLT3mut leukemia mice [58]. In a similar study, apoptosis is potently and synergistically triggered in FLT3‐ITD AML cell lines and primary patient samples when the FLT3 inhibitors gilteritinib or midostaurin are combined with the Bcl‐2 inhibitor venetoclax. The FLT3 inhibitors downregulate Mcl‐1, increasing the activity of venetoclax. Moreover, in vivo results show that the administration of gilteritinib and venetoclax together has therapeutic potential [55].

In individuals of various age groups, including newborns, kids, and grownups diagnosed with *KMT2A*‐r‐ALL, as well as children with *KMT2A*‐r‐AML, there are notable increases in the levels of gene expression of *SYK*, *FLT3*, *JAK2/JAK3*, *BTK*, and several SRC family protein tyrosine kinases (*PTK*). Additionally, in the case of grownups with *KMT2A*‐r‐AML, distinct alterations in gene expression were observed, particularly increases in JAK family kinase TYK2, SYK, and the SRC family kinases FGR and HCK [59]. This offers tyrosine kinases, among other signaling pathways in *KMT2A*‐r acute leukemias as possible treatment targets for beating drug resistance in cancer.

### 5.5 Proteasome Inhibition

Proteolysis‐targeting chimera (PROTAC) degraders have been investigated as a possible therapeutic alternative to inhibit canonical and non‐canonical oncogenic activity [60]. In a recent study, a new and extremely powerful EZH2 PROTAC degrader, MS8847, was found to trigger antiproliferative effects and degradation of EZH2 in *KMT2A*‐r‐AML cells [61]. Targeting the proteasome with drugs like bortezomib can disrupt the degradation of specific proteins, including tumor suppressors, leading to apoptosis in *KMT2A*‐r leukemia cells. The proteasome inhibitors carfilzomib and bortezomib have shown clinical efficacy in treating ALL and are authorized for the treatment of multiple myeloma and mantle cell lymphoma [40]. It is observed that bortezomib effectively restrained cell growth and reduced the formation of colonies in the leukemia cells of humans and also murine leukemia cells. Additionally, LSC (Leukemia Stem Cell) frequency and function were reduced by bortezomib, hindered disease advancement, and prolonged the general survival in mice with MLL‐AF9‐rearranged leukemia. Furthermore, bortezomib resulted in a reduction in the proportion of human LSC (characterized by CD34+ CD38‐ phenotype) and increased the general survival in mice engrafted with AML blasts, particularly in the NOD/SCID‐IL2Rγ (NSG) model [62]. Similarly, bortezomib demonstrated a dosage‐dependent increase in the development of miR‐29b in AML cells in vitro, leading to reduced levels of DNA methyltransferases (Dnmts), inhibited cell proliferation, and an enhanced rate of programmed cell death (apoptosis). However, in vivo administration of bortezomib against dKI AML showed no efficacy. In contrast, when administered as a single agent in vivo, liposome‐encapsulated bortezomib successfully reversed the reduction of miR‐29b expression and provoked a long‐term remission free of illness lasting 90 days in 80% of dKI AML mice. Notably, these mice initially had a great burden of leukemia at the beginning of the treatment and exhibited no signs of relapse upon autopsy [63]. Therefore, when used alone, liposomal bortezomib effectively eliminates Mll(PTD/wt):Flt3(ITD/wt) AML in mouse models. This approach holds significant promise as a potent and possibly therapeutic approach for human diseases that are at risk.

On the contrary, although the proteasome inhibitor, carfilzomib, demonstrated in vitro synergy with a number of traditional chemotherapeutic agents such as vincristine, dexamethasone, daunorubicin, L‐asparaginase, and 4‐hydroperoxycyclophosphamide, it failed to produce survival advantage for either carfilzomib monotherapy or combined therapy with multiple chemotherapeutic agents in both high or low disease burden [64]. This underscores that although proteasome inhibitors are crucial in treating various blood‐related cancers, their effectiveness in the in vitro setting may not always align with their real in vivo benefits. It emphasizes the significance of validating their performance in living organisms before considering them for clinical application. Thus, more clinical explorations are needed.

### 5.6 Immunotherapies

Immunotherapeutic strategies, such as chimeric antigen receptor (CAR) T‐cell therapy and antibody‐drug conjugates, are being explored to target specific cell surface markers present in *KMT2A*‐r leukemia cells. CD19‐targeted CAR‐T cells (e.g., tisagenlecleucel) have been used in B‐cell ALL [65]. Moreover, drug‐antibody conjugates such as inotuzumab ozogamicin target CD22 and are documented in treating relapsed or refractory ALL [66, 67]. In a clinical study, authors assessed the treatment outcome of kids and youthful adults with relapsed/refractory CD19+ ALL/lymphoblastic lymphoma treated on 5 CD19‐directed CAR T‐cell (CTL019 or humanized CART19) clinical trials or with commercial tisagenlecleucel. According to the results, the complete remission rate was 94%, with no disparity between strata according to their risk categories. Additionally, there was no distinction between overall survival (OS) and relapse‐free survival [65]. Thus, CTL019, huCART19, and tisagenlecleucel successfully achieved long‐lasting remissions in various cytogenetic categories [65]. Patients who have relapsed or are refractory and have cytogenetics that are high‐risk, such as those with *KMT2A*‐r iALL, showed a notable increase in the likelihood of overall survival (OS) and relapse‐free survival (RFS) at the two‐year mark [65].

Tisagenlecleucel and huCART19 effectively treat diseases of the central nervous system (CNS) and maintain long‐lasting periods of remission in young patients with lymphocytic lymphoma or B‐cell ALL that has relapsed or is refractory. Importantly, they do so without significantly raising the risk of severe neurotoxicity [68]. However, it is advisable to exercise caution regarding the timing of disease management and therapy to minimize this potential harm. These initial results encourage the utilization of these CAR T‐cell therapies for patients dealing with CNS‐relapsed or refractory B‐cell ALL. Several investigations have shown the encouraging potential of immunoproteasome inhibitors KZ‐616 and M3258 in ALL [40].

### 5.7 Synthetic Lethality

Identifying synthetically lethal genes with *KMT2A* fusion genes has opened new avenues for therapeutic development. Synthetic lethality‐based therapies target the vulnerabilities specific to *KMT2A*‐r leukemias. For example, drugs that inhibit poly‐ADP ribose polymerase (PARP), such as olaparib target synthetic lethality with mutations in genes responsible for DNA repair. As a cancer treatment, PARP inhibitors prevent the PARP from carrying out its repair activity in cancer cells, causing the cells to die [69, 70]. Combining olaparib with DNMT inhibitors or chemotherapy leads to a significant decrease in the creation of colonies of cells or cell growth and induces the blocking of the cell cycle and programmed cell death in MLL‐AF9 leukemic cells when compared to using these agents individually [71]. Another study found that AML fueled by repressive transcription factors, such as PML‐RARα fusion oncoproteins (resulting from *PML‐RARA*) and AML1‐ETO (resulting from the fusion oncogene *RUNX1‐RUNX1T1*) display high sensitivity to inhibition of PARP. This heightened sensitivity is attributed to their decreased production of critical homologous recombination (HR)‐related genes and their impaired response to damaged DNA (DDR). In contrast, leukemia caused by *KMT2A* fusions with strong transactivation capabilities demonstrates DDR proficiency and resistance to PARP inhibition [72]. Moreover, while the blockage of HOXA9, an KMT2A downstream target, sensitizes *KMT2A*‐r leukemia to PARP inhibitors and impairs DDR, the overexpression of HOXA9 rather confers PARP inhibitor resistance to *AML1‐ETO* and *PML‐RARα* transformed cells [72]. Furthermore, PARP inhibitors olaparib and Temozolomide specifically cause leukemic cells that are positive for *TCF3‐HLF* to become toxic [73]. These observations present an innovative therapeutic strategy for combating refractory leukemia, including those with *KMT2A*‐r in both AML and ALL.

### 5.8 Stem Cell Transplantation

In high‐risk cases, transplanting allogeneic stem cells is thought to be a curative approach. This approach involves replacing the diseased individual's hematopoietic system with healthy cells from a donor. Stem cell transplants may involve genetically matched donors and recipients to minimize the chances of graft‐versus‐host disease. In a clinical study of patients with *MLL* /*KMT2A* rearrangement, it was found that solid tumor therapy‐related AML (t‐AML), MLL t‐AML, had bad prognosis in comparison to non‐MLL t‐AML and MLL de novo AML, but allogeneic hematopoietic stem cell transplantation (allo‐HSCT) overcame the poor prognosis of MLL t‐AML [74]. According to a related study, individuals treated with allo‐HSCT have a better prognosis and higher survival rates than those treated with chemotherapy alone when it comes to treating *KMT2A*‐r‐AML [75]. However, *KMT2A*‐r‐AML patients who experienced thrombocytopenia at the beginning (< 50 × 10^9^) had extremely poor OS and disease‐free survival (DFS) [75]. There is also a case report of a person with 11q23/*KMT2A*‐r‐AML who had a third allo‐HSCT successfully after undergoing treatment with azacitidine and venetoclax. The result indicates that a combined therapy of allo‐HSCT with azacitidine and venetoclax might be a successful strategy for relapsed and refractory *KMT2A*‐r‐AML [76]. Other combined therapies, such as venetoclax‐containing myeloablative conditioning (MAC) allo‐HSCT regimens, are reported to be safe, practical, and efficient for patients with high‐risk AML [77]. Despite these promising outcomes, clinical data on this subject are largely lacking, and the few existing studies are primarily small‐scale. More large‐scale clinical studies are needed.

## 6 Clinical Trials

Extensive clinical trials are currently evaluating numerous therapeutic targets in *KMT2A*‐r leukemia from the Clinical Study Database (ClinicalTrials.gov), thereby affording patients access to innovative and groundbreaking therapeutic interventions. Participation in clinical trials can provide opportunities to benefit from the latest therapeutic strategies. In *KMT2A*‐r leukemias, menin is a protein essential for transforming blood cells into cancer cells. Menin inhibition leads to the death of leukemia cells by triggering differentiation and apoptosis [32, 78]. NPM1 is another protein that is important in the development of leukemia. It binds to DNA at specific sites that KMT2A also occupies. Inhibiting menin triggers the degradation of NPM1, which leads to a reduction in the expression of genes essential for leukemia cells to grow and survive [79]. Therefore, disrupting the menin‐KMT2A axis is a promising treatment approach for tackling leukemias driven by *KMT2A* rearrangements and *NPM1* mutations. There are at least six distinct menin‐KMT2A inhibitors that are currently being evaluated in clinical trials as first‐ and second‐line monotherapy for acute leukemias (DSP‐5336, BMF‐219, DS‐1594, JNJ‐75276617, ziftomenib, and revumenib) [80]. for two of these inhibitors, revumenib and ziftomenib, which have shown promising results [80]. In an efficacy analysis of the phase 1 clinical trial of revumenib (SNDX‐5613), In *KMT2A*‐r or *NPM1*‐mutant acute leukemia, the authors report a 30% rate of full remission or complete remission with partial hematologic recovery and a low frequency of Grade 3 or higher treatment‐related side events [2]. This clinical data established menin inhibition as a treatment approach for susceptible subtypes of acute leukemia.

In a risk‐stratified therapeutic study of babies suffering from *KMT2A*‐r ALL, patients were given high‐dose cytarabine along with modified chemotherapy, and only high‐risk patients were eligible for HSCT. Newborns with *KMT2A*‐r ALL (Intermediate + high risk) had a 3‐year event‐free survival (EFS) rate of 66.2%, while newborns with germline *KMT2A* (*KMT2A*‐g) ALL (low risk) had a rate of 93.3%. For patients at moderate risk, the 3‐year EFS rate was 94.4%, while for those at high risk, it was 56.6% [81]. This indicates the importance of risk stratification and the implementation of rigorous chemotherapy in leukemia interventions, which should be considered in future trials. In an international phase III randomized study that investigated whether myeloid‐style consolidation chemotherapy is better than lymphoid style, patients receive either the lymphoid course (low‐dose cytosine arabinoside [araC], cyclophosphamide (IB)), 6‐mercaptopurine or experimental myeloid courses (araC, daunorubicin, etoposide (ADE) and mitoxantrone, araC, etoposide (MAE)). It was concluded that early intensification of treatment with post‐induction myeloid‐type chemotherapy did not result in better outcomes for infants with ALL in contrast to the standard lymphoid‐type chemotherapy. The results of the Interfant‐06 trial for newborns with ALL were not better than those in the Interfant‐99 trial, which was conducted earlier [82]. In other words, adding more and more potent chemotherapy drugs to the early treatment of infant ALL did not make a difference in how well the children did. This is important information for doctors and parents to know when making decisions about how to treat infants ALL. Moreover, this provides an avenue for further exploration to consolidate apparent contradictions and improve existing and evolving therapies. The therapeutic targets expanded above are listed in Table 1 as a summary.

**TABLE 1:** Therapeutic targets of *KMT2A*‐r acute leukemia in experimental and clinical studies.

Type of study/disease	Therapeutic agent	Target/mechanism	Key outcome	Reference
Experimental with MLL–AF4 cells and male NSG mice	ONX‐0914 LU‐102	ONX‐0914 inhibits LMP7 (ß5i) and LMP2 (ß1i) sites of the immunoproteasome, and LU‐102 inhibits proteasome ß2 sites in ALL	Synergistic cytotoxicity, delayed development of orthotopic ALL xenograft tumors in mice, and lines of T‐cell ALL sensitivity to ONX‐0914	[40]
Experimental with *KMT2A*‐r‐AML and *KMT2A*‐r‐ALL cells (MOLM‐13, THP‐1, MV4‐11, and SEM)	HDAC inhibitor I1	Regulates epigenesis by encouraging the differentiation of cells, combined with cell cycle blockage at the G0/G1 phase	Inhibits the proliferation and the ability of the cancer cells to form colonies	[43]
Experimental with *KMT2A*‐r and leukemic stem‐like cells (Kasumi‐1, KG‐1, MOLM‐13, and THP‐1)	HDAC inhibitor I3	Regulates epigenesis by encouraging the differentiation of cells combined with cell cycle blockage at the G0/G1 phase and inhibition of the VEGF‐MAPK signaling pathway	Efficient antiproliferative activity via enhancing cell differentiation, coupled with cell cycle exit at G0/G1	[45]
Experimental with AML cell lines (Kasumi‐1, KG‐1, MOLM‐13, and NB4)	HDAC inhibitor I13	On leukemic cells, the activation of the antigen processing and presentation pathway was probably determined by epigenetic regulation through HDAC inhibition.	Causes leukemic stem‐like cells, M2 subtype AML cells with t(8;21) translocation, and M3 and M5 subtype AML cells to differentiate.	[46]
Experimental with murine acute myeloid leukemias (MLL‐AF9); Nras(G12D); PML‐RARα APL cells and Eμ‐Myc lymphoma in vitro and in vivo	HDAC1/2‐specific inhibitor RGFP233; HDAC3 inhibitor RGFP966	Inhibition of individual or multiple Hdacs	Co‐depletion of Hdac1 with Hdac2 facilitates a pro‐apoptotic response that is robust; HDAC3 inhibitor RGFP966 decreased Eμ‐Myc lymphoma proliferation and differentiation in APL	[44]
Experimental with MLL‐positive BCP‐ALL, SEM and RS4;11 cell lines	Hypomethylating agents azacitidine and decitabine with/without cytostatic drugs cytarabine and doxorubicin	Inhibition of DNA hypermethylation	Significant reduction of proliferation by provoking the arrest of the cell cycle in G0/G1 phase and programmed cell death; best anti‐cancer effect observed at the simultaneous exposure to HMA and cytostatic drugs	[47]
Experimental with B‐ALL cells in vitro	CDK7 inhibitor THZ1	Induces the programmed cell death of B‐ALL cells by disrupting cellular metabolism mediated by c‐MYC	Arrests the cell cycle, impedes cellular metabolism, and prevents the formation of cellular metabolic intermediates	[51]
Experimental with human NPM1mut and FLT3mut AML cells; mice with *KMT2A*‐r FLT3mut leukemia	FLT3 inhibitor quizartinib and Menin‐KMT2A inhibitor MI‐503	Combined inhibition of FLT3 phosphorylation and Menin‐KMT2A interaction	Considerably greater responses to menin and FLT3 inhibition when combined than a single‐drug; lowers the burden of leukemia and longer mice survival	[58]
Experimental with FLT3‐ITD AML in vitro and in vivo	FLT3 inhibitors midostaurin or gilteritinib with Bcl‐2 inhibitor venetoclax	Combined inhibition of FLT3 and Bcl‐2	Simultaneous downregulation of Mcl‐1 and inhibition of Bcl‐2 lead to synergistic induction of apoptosis.	[55]
Experimental with MLL‐AF9‐transformed leukemic mice, NOD/SCID‐IL2Rγ (NSG) mice	Proteasome inhibitor bortezomib	Decreases CDK6 by obstructing NF‐ĸB recruitment to the CDK6 promoter	Selective targeting of LSC in *KMT2A* rearrangements, suppressing the proliferation of cells and the formation of colonies and increasing overall survival in mice	[62]
Experimental with Mll(PTD/wt):Flt3(ITD/wt) AML mice; dKI AML mice; AML cells	Proteasome inhibitor bortezomib (liposomal bortezomib)	Increases miR‐29b expression and decreases Dnmt1, 3a, and 3b	Increases apoptosis and decreases proliferation in AML blasts in vitro; long‐term remission that is disease‐free in 80% of dKI AML mice	[63]
Experimental with human MLL‐AF9 leukemic cell line and primary mouse leukemia cells	PARP inhibitor olaparib with chemotherapy agents or DNMT inhibitors	Increased number of cells with > 10 γH2AX DNA damage foci and double‐stranded breaks	Decreased colony formation and cell development, with enhanced cell cycle block and apoptosis	[71]
Experimental with ALL and AML cell lines and female NSG mice	PARP inhibitor olaparib and temozolomide	Targets DNA damage or homologous recombination repair (HRR)	Selective induction of cytotoxicity in TCF3‐HLF–positive leukemic cells	[73]
Clinical study in patients with *KMT2A*‐r‐AML with solid tumor	Allo‐HSCT	Hematopoiesis	Eliminates the bad prognosis in *KMT2A*‐r solid tumor therapy‐related AML	[74]
Clinical study on *KMT2A*‐r‐AML patients	Allo‐HSCT	Hematopoiesis	Allo‐HSCT exhibits a better prognosis and more prolonged survival than chemotherapy	[75]
Clinical evaluation of safety and efficacy in high‐risk AML patients	A MAC regimen with venetoclax and allo‐HSCT	Hematopoiesis	For patients with high‐risk AML, adding venetoclax to a MAC allo‐HSCT is practical, secure, and efficient.	[77]
Preclinical effectiveness evaluation of carfilzomib, a selective proteasome inhibitor, in vitro and in vivo for neonates with *KMT2A*‐r ALL	Proteasome inhibitor carfilzomib	Proteasome inhibition	In vitro synergy between carfilzomib and several conventional chemotherapeutic agents but no survival advantage in vivo	[64]
Patients with relapsed/refractory CD19+ ALL/lymphoblastic lymphoma treated with CTL019 or huCART19 clinical trials or with commercial tisagenlecleucel	CAR T‐cell therapies CTL019, huCART19, and tisagenlecleucel	Immunotherapy	Overall complete remission rate of 94% and no disparity in relapse‐free survival across cytogenetic categories	[65]
Clinical safety assessment and activity of CAR T‐cell therapy in patients with a history of CNS relapsed or refractory B‐cell ALL	CAR T‐cell therapies tisagenlecleucel and huCART19	Immunotherapy	Clearance of CNS disease and maintaining durable remissions in patients with CNS relapsed or refractory B‐cell ALL or lymphocytic lymphoma	[68]
Second phase clinical trial on *KMT2A*‐r leukemia	Combined therapy of bortezomib and vorinostat	Proteasome and HDAC inhibition	The response rate could not be computed since the trial was stopped before the maximum tolerated dose was established.	NCT02419755
Phase I/II clinical trial on relapsed/refractory AML/ ALL with/without *KMT2A* rearrangement or *NPM1* mutation	DSP‐5336	Menin‐KMT2A inhibition	Ongoing and recruiting	NCT04988555
Phase I/II clinical trial on *MLL/KMT2A* gene rearrangement or NPM1 mutation	SNDX‐5613/cobicistat	Menin‐KMT2A interaction	Ongoing and recruiting	NCT04065399
Phase I clinical trial on KMT2A‐r acute leukemia with or without gene mutations	Ziftomenib, venetoclax, azacitidine, daunorubicin, and cytarabine	Evaluate the initial antileukemic activity, safety, and tolerability	Ongoing and recruiting	NCT05735184
Phase Ib clinical trial on AML with *KMT2A* rearrangement and AML with NPM1 mutation	Cytarabine, daunorubicin, and revumenib	Menin inhibition	Ongoing and recruiting	NCT05886049

Abbreviations: allo‐HSCT, allogeneic hematopoietic stem cell transplantation; AML, acute myeloid leukemia; APL, acute promyelocytic leukemia; B‐ALL, B‐cell acute lymphocytic leukemia; CDK6, cyclin‐dependent kinase 6; Dnmt, DNA methyltransferases; HMA, hypomethylating agents; huCART19, humanized CD19‐directed CAR; ITD, internal tandem duplication; MAC, myeloablative conditioning.

## 7 Conclusion and Perspective

*KMT2A*‐r acute leukemia is linked to a poor treatment outcome. This subtype of leukemia is often resistant to conventional chemotherapy, leading to decreased rates of remission and greater rates of relapse compared to other leukemia types. The aggressive nature of *KMT2A*‐r acute leukemia contributes to its challenging treatment landscape. Typically, treatment strategies for *KMT2A*‐r acute leukemia involve vigorous chemotherapy regimens, transplantation of stem cells, and specific therapies. While some patients may achieve remission, long‐term survival rates remain relatively low, especially in cases of adult *KMT2A*‐r acute leukemia. Research is ongoing to develop more effective and targeted treatments for *KMT2A*‐r acute leukemia, but it remains a challenging disease to manage. Prognosis and treatment outcomes may vary depending on factors like age, specific *KMT2A* rearrangement, and the presence of other genetic mutations.

Regardless, the perspective and prospects of *KMT2A*‐r acute leukemia research are promising and include several key aspects. Firstly, researchers are actively investigating targeted therapies specific to *KMT2A*‐r acute leukemia. These therapies aim to disrupt the KMT2A fusion proteins, which are essential to the illness. Identifying and developing drugs that target these fusion proteins could significantly improve treatment outcomes. Secondly, immunotherapeutic approaches, such as CAR T‐cell therapy, hold potential in treating *KMT2A*‐r leukemia. CAR T‐cell therapies are being explored to specifically target and eliminate leukemia cells, potentially leading to more effective treatments. Moreover, advances in genomic sequencing continue to provide a deeper understanding of the genetic alterations associated with *KMT2A*‐r leukemia. This knowledge can guide the development of precision medicine strategies and the identification of new therapeutic targets. In addition, researchers are exploring combinations of existing treatments, such as chemotherapy, epigenetic modifiers, and targeted therapies, to enhance the prognosis for patients with *KMT2A*‐r leukemia. Combinatorial approaches aim to maximize treatment efficacy while minimizing side effects. For example, a study reports that the strongest antiproliferative effects in *KMT2A*‐r‐ALL are observed when the cancer cells are simultaneously exposed to hypomethylating agents (azacitidine and decitabine) and cytostatic drugs (cytarabine and doxorubicin). Cytarabine was used to provoke the most powerful synergistic effects of hypomethylating drugs [47]. This opens the front for further clinical exploration of combined therapies.

Identifying reliable biomarkers for *KMT2A*‐r leukemia can aid in early detection, risk stratification, and treatment monitoring. Biomarker research is essential for developing personalized treatment plans; thus, there is a need for more future studies in this direction. Due to the essential role of clinical trials in testing and validating new treatment approaches, more trials are needed in future studies. This contributes to advancing the field and subsequent translation in the clinical setting. Improving the overall care and support for *KMT2A*‐r leukemia patients is a crucial aspect of research. This includes enhancing supportive care, managing side effects, and addressing the unique needs of this patient population. Finally, future studies should consider collaborative efforts between researchers and institutions worldwide to accelerate progress in *KMT2A*‐r leukemia research. Sharing knowledge, data, and resources is vital for significantly advancing this critical disease.

In summary, the future of *KMT2A*‐r acute leukemia research is marked by a deepening understanding of the disease's biology, a focus on personalized treatments, immunotherapies, genomics, and innovative combinations of therapies. The ultimate goal is to improve patient outcomes, reduce the burden of illness, and improve the affected people's quality of life. Research and clinical exploration in this field are still ongoing and hold the prospect of improving therapeutic approaches and patient outcomes.

## Author Contributions

**Lei Yin:** writing – original draft (lead). **Lin Wan:** writing – review and editing (equal). **Youjian Zhang:** investigation (equal), resources (equal), validation (equal), visualization (equal). **Shenghao Hua:** formal analysis (equal), validation (equal), visualization (equal). **Xuejun Shao:** writing – review and editing (equal).

## Ethics Statement

The authors have nothing to report.

## Consent

The authors have nothing to report.

## Conflicts of Interest

The authors declare no conflicts of interest.

## Data Availability

The authors have nothing to report.
